# Safety assessment and sustainability of consuming eggplant (*Solanum melongena* L.) grown in wastewater-contaminated agricultural soils

**DOI:** 10.1038/s41598-022-13992-7

**Published:** 2022-06-13

**Authors:** Khalid H. Alamer, Tarek M. Galal

**Affiliations:** 1 Biological Sciences Department, Faculty of Science and Arts, King Abdulaziz University, Rabigh, 21911 Saudi Arabia; 2 Botany and Microbiology Department, Faculty of Science, Helwan University, Cairo, 11790 Egypt; 3 Biology Department, College of Sciences, Taif University, P.O. Box 11099, Taif, 21944 Saudi Arabia

**Keywords:** Agroecology, Ecology, Health care

## Abstract

Vegetables cultivated on contaminated agricultural soils are being consumed by the public, and consequently cause serious health concerns due to contaminants' dietary intake. The current study examines the safety and sustainability of eating eggplant (*Solanum melongena*) by looking into the possibility of heavy metals translocation from polluted soils to the edible sections, as well as the health hazards that come with it. Soil and eggplant samples were taken from three contaminated and other three uncontaminated farms to estimate their chemical constituents and plant growth properties. Based on the pollution load index data, the contaminated soils were highly polluted with Fe, Cu, Pb, and Zn; and relatively polluted with Cr, Mn, Cd, Mn, Co, and V. Under contamination stress, the fresh biomass, dry biomass, and production of eggplant were significantly reduced by 41.2, 44.6, and 52.1%, respectively. Likewise, chlorophyll a and b were significantly reduced from 1.51 to 0.69 mg g^−1^ and 1.36 to 0.64 mg g^−1^, respectively. The uncontaminated plant shoots had the highest quantities of N, P, and proteins (1.98, 2.08, and 12.40%, respectively), while the roots of the same plants had the highest K content (44.70 mg kg^−1^). Because eggplant maintained most tested heavy elements (excluding Zn and Pb) in the root, it is a good candidate for these metals' phytostabilization. However, it had the potential to translocate Mn and Zn to its shoot and Pb, Cr, Mn, and Zn to the edible fruits indicating its possibility to be a phytoextractor and accumulator of these metals. Cd, Cu, Ni, Pb, Mn, and Co quantity in the edible sections of eggplant grown in contaminated soils exceeded the permissible level for normal plants, posing health hazards to adults and children. For safety issues and food sustainability, our investigation strongly recommends avoiding, possibly, the cultivation of eggplant in contaminated agricultural lands due to their toxic effects even in the long run.

## Introduction

Food sustainability and safety is a global public issue that has drawn scientists' attention to the health risks connected with consuming tainted foodstuffs^1^. Unprecedented population growth and urbanization generate tremendous quantities of wastewater worldwide; their safe disposal is one of the common environmental concerns^2^. In developing countries, wastewater is an alternative irrigation source commonly used to maximize the yield of high-quality food crops in urban areas^3^. Wastewater is the main contributor to soil contamination; and thus, irrigating food crops with such water is a serious issue as these effluents are heavily loaded with toxic heavy metals^4,5^. Although fertilizers and pesticides are important for crop growth and production, their extensive use may leave residues on food products, which can potentially cause human health risks^6^.

Various pollutants are widely spread in the environment; heavy metals are of particular interest because of their bioaccumulation potential and harmful impacts on ecosystems^7^. Heavy metals are the most important concern in terms of vegetable growth and yield in contaminated agricultural fields all over the world^8^. They degrade soil, reducing vegetable quality, output, and food safety, thereby making vegetable cultivation unsustainable^9^. The most serious and pervasive agricultural concerns in emerging countries are heavy metal-contaminated soils^10^. Their accumulation in crop plants through contaminated wastewater irrigation can cause devastating impacts on consumer health^11^. The increased level of heavy metals because of natural processes or anthropogenic activity has exacerbated the food security threats for the world's increasing population^12,13^. Cultivating vegetables in polluted agricultural fields increases the danger of toxic metal poisoning, which can be harmful to human health^14^.

Vegetables grown with wastewater irrigation from sewage and industrial effluents are consumed by the general people, posing serious health risks because of heavy metal accumulation through dietary consumption of these polluted food plants^15,16^. After tomato, potato, and pepper, *Solanum melongena* L. (eggplant) is one of the common commercially economic solanaceous crops^17^. It is a high-yielding and biomass-productive fruit crop, which is well-adapted to hot and wet environments^18^. Eggplant has numerous health benefits, including its function in preventing chronic diseases^19^, as well as the effects of its phenolic components on human health, such as cardioprotective, anti-carcinogenic, antioxidant, anti-inflammatory, and anti-diabetic qualities^20^. Worldwide, more than 1,800,000 ha produce about 50 million tons of eggplant^21^, Accessed 03.08.2017). The production of eggplant is common in a few countries; 29.5 million tons in China, which is the top producer, followed by 13.5 million tons in India, 1.2 million tons in Egypt, 0.85 million tons in Iran, and 0.82 million tons in Turkey^20^.

In most developing countries, water scarcity is the primary worry^22^. According to the International Water Management Institute, 1.8 billion people would face severe water shortages by 2025^23^. Thus, using nonconventional irrigation (e.g., wastewater) is an indispensable resource of irrigation water to mitigate the pressure on freshwater^24^. In this context, we hypothesize that the cultivation of vegetable crops in wastewater-irrigated agricultural soil may threaten food safety and human health. Consequently, investigating plant-heavy metals content is crucial to mitigate their concentration in crop plants and avoid their toxicity^25^. Therefore, the goal of this study was to assess the extent of heavy metals translocation from wastewater-irrigated soil to the edible sections of *Solanum melongena* (eggplant) to determine its health safety and sustainability for the public consumption.

## Materials and methods

### Study sites

**Figure 1:**
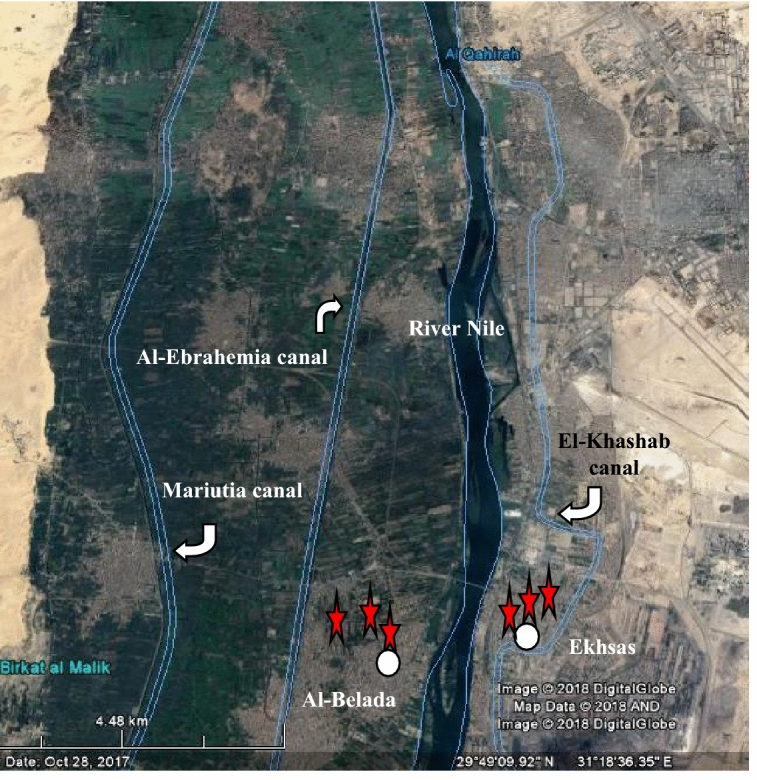
The study sites shown on a map of south Greater Cairo. 29° 49ʹ 09.92ʺ N and 31° 18ʹ 36.35ʺ E. Source: Google earth 18 October 2017. Modified by the author using Microsoft Word version 2007.

The study area lies in an urbanized region in Greater Cairo and comprised two sites; one of them is contaminated and receives industrial and municipal wastewater, and agricultural drainage (Fig. 1). It is situated on the eastern bank of the Nile in the Ekhsas neighborhood (29° 47′ 29.31′′ N–31° 18′ 38.05′′ E), south Cairo Province, and houses 42.6 percent of Greater Cairo's 18.29 million consumers^26^. The soils of this site suffer from high water levels, which may lead to desertification. However, another site is uncontaminated (control) and irrigated from the River Nile water through Al-Ebrahemia canal, which in turn receives no discharge of industrial or municipal wastes. It was located on the River Nile's western side et al.-Belada village (29° 42′ 11.06′′ N–31° 15′ 52.51′′ E), south Giza Province. The study sites extend along the Nile Valley, and thus their soils are alluvial. The research area's climate was defined by a mean annual temperature of 21.08 degrees Celsius, a mean annual relative humidity of 52.68 to 56.08 percent, and a yearly average rainfall of 1.67–2.13 mm per year.

### Plant sampling and growth measurements

Samples of eggplant plants were taken from 6 different farms (about 2 acres each) and equally distributed in the contaminated and uncontaminated sites. Collected plants were the same cultivar and in the same growth stage (fruiting) and grown in the same conditions of climate and soil type. Voucher specimens were identified by the author (Tarek M. Galal) and deposited in Helwan University Herbarium, Cairo, Egypt. At each cultivated farm, 10 quadrats (2 × 2 m each) were randomly chosen, to collect the eggplant samples. By counting the number of individuals in each quadrat, plant density, as the number of individuals per 100 m^2^, was obtained, and plants were then collected and transported to the laboratory for further analysis. Plant samples were cleaned twice with distilled water and once with tap water to eliminate any dirt or dust. After measuring the length of the roots and shoots, as well as the count of the leaves per individual, the samples were sorted into roots, shoots, and fruits (edible parts). To measure fresh and dry biomass (weight/unit area), the separated shoots were weighed before and after oven drying at 105 °C until constant weight. Besides, the fruit's fresh weight was used to estimate fruit production.

### Plant analysis

#### Chemical analysis

For chemical analysis, three composite oven-dried eggplant roots, shoots, and fruits (N = 54) were gathered from each of the contaminated and uncontaminated farms and processed into a powder in a metal-free plastic mill. Kjeldahl and molybdenum blue procedures were used to estimate total N and total P, respectively, while to estimate K, a flame photometer (CORNING M410) was used. Umbriet et al.^27^ and Lowry et al.^28^ procedures were used to calculate total soluble sugars (carbohydrates) and total soluble proteins, respectively. The acid digestion method was used to estimate the heavy metals concentration of sampled plant parts^29^. A ground sample of 1 g was digested in 20 mL of a tri-acid mixture of HNO_3_:H_2_SO_4_:HClO_4_ (5:1:1, *v/v/v*) until a transparent color appeared, then the digested plant was filtered and diluted with double-distilled water to 25 mL. Shimadzu AA-6300 (Shimadzu Co. Ltd., Kyoto, Japan) Spectrophotometer was used to estimate the amounts of Co, Fe, Cu, Ni, Mn, Pb, Cd, Zn, Cr, and V^29^. The detection limits for heavy metals were 9.0 for Co, 5.0 for Fe, 1.5 for Cu, 6.0 for Ni, 0.8 for Cd, Mn, and Zn, 15.0 for Pb, 3.0 for Cr, and 2.0 for V (in µg/l). The confidence level for all detection thresholds was 95% (3 standard deviations).

##### Quality control and assurance

A recognized reference sample (SRM 1573a, tomato leaves) was utilized to assess heavy metals accuracy, with recovery rates ranging from 94.8 to 103.7%. The digestion and analysis of the certified reference material were carried out with the same methods applied to the eggplant tissues. Three replicates were used for heavy metals digestion and determination. The measured heavy metals concentration was compared with those of the certified value to estimate the accuracy percentage.

#### Pigment content

From each quadrat in the contaminated and uncontaminated areas, three eggplant leaves were combined to generate 3 composite samples (N = 18) for each farm. Chlorophyll a, b, and carotenoids were prepared from 2 g fresh leaves by immersing them in 50 percent (v/v) acetone overnight at 4 °C in full darkness. The extract was then compared to aqueous acetone, as a blank, spectrophotometrically at three wavelengths: 663, 644, and 453 nm. Each pigment's content (mg g^−1^ fresh wt.) was calculated as follows^29^:

1 $$ {\text{Chl}}.{\text{ a }} = { 1}0.{\text{3 A}}_{{{663}}} {-} \, 0.{\text{918 A}}_{{{644}}} $$

2 $$ {\text{Chl}}.{\text{ b }} = { 19}.{\text{7 A}}_{{{644}}} {-}{ 3}.{\text{87 A}}_{{{663}}} $$

3 $$ {\text{Carotenoids }} = { 4}.{\text{2 A}}_{{{453}}} {-} \, \left( {0.0{\text{264 chl}}.{\text{ a }} + \, 0.{\text{426 chl}}.{\text{ b}}} \right), $$

where A represents the absorbance.

### Soil analysis

From each farm in the uncontaminated and contaminated areas, three composite sub-surface soil samples (N = 18) were taken from the eggplant rhizosphere. The electrical conductivity (EC) and pH value of 1:5 *w/v* soil–water extracts were determined using a conductivity meter 60 Sensor Operating Instruction Corning and a glass electrode pH meter (Model 9107 BN, ORION type), respectively. Nutrient elements (N, P, and K) and heavy metals (Co, Fe, Cu, Ni, Mn, Pb, Cd, Zn, Cr, and V) concentrations were determined using the same procedures in plant analysis^29^.

### Data analysis

#### The pollution load index (PLI)

The PLI rates each heavy metal's soil pollution as follows:

4 $$ {\text{PLI }} = {\text{ C}}_{{\text{c}}} /{\text{C}}_{{\text{u}}} $$

where C_c_ and C_u_ are the heavy metals content in contaminated and uncontaminated soils, respectively^30^. The soil was categorized according to the PLI following Lu et al.^31^: uncontaminated: PLI = 0–1; uncontaminated- relatively contaminated: PLI = 1–2; relatively contaminated: PLI = 2–3; relatively -highly contaminated: PLI = 3–4; highly contaminated: PLI = 4–5; and very highly contaminated: PLI > 5.

#### Transfer factors (TF)

The transfer factor (TF) is a good indicator to represent heavy metal transmission from the soil to the various plant organs. It assumes a linear link between heavy metal concentrations in soil and plants. The TF is used to look at a plant's ability to acquire heavy metals in its underground roots and then translocate them to its aboveground shoot. According to Eid et al.^32^, it was computed as follows:

5 $$ {\text{TF}}_{{{\text{root}}}} = {\text{C}}_{{{\text{ro}}}} /{\text{C}}_{{{\text{so}}}} $$

6 $$ {\text{TF}}_{{{\text{shoot}}}} = {\text{C}}_{{{\text{sh}}}} /{\text{C}}_{{{\text{ro}}}} $$

7 $$ {\text{TF}}_{{{\text{fruit}}}} = {\text{C}}_{{{\text{fr}}}} /{\text{C}}_{{{\text{ro}}}} $$

where, C_so_, C_ro_, C_sh_, and C_fr_ are the element concentration (mg kg^−1^) in the soil, root, shoot, and fruit, respectively.

#### Health risk assessment

The daily intake of metals (DIM) for both children and adults was calculated following^33^

8 $$ {\text{DIM }} = \, \left( {{\text{C }} \times {\text{ F }} \times {\text{ D}}} \right) \, /{\text{ B}} $$

where C is the plant concentration of heavy metal (mg kg^−1^), F is a factor (0.085) for converting fresh to dry weight^34^, D is the daily vegetable requirement (0.345 and 0.232 kg/person^/^day) for children and adults, respectively, and B is the mean body weight (32.7 and 55.9 kg) for children and adults^22^. Furthermore, the estimated crop exposure / the reference oral dose ratio was used to calculate the health risk index (HRI) for local consumers of contaminated plants^32^. When the HRI exceeded one, it implies that the consumers' health is at risk^35^.

### Statistical analysis

A paired-sample t-test was used to compare the differences in soil and plant characteristics between uncontaminated and contaminated areas. However, using SPSS software^36^, ANOVA 1 was used to estimate the considerable nutrients and heavy metals variation among the different plant organs. Duncan’s multiple range test was used to assess significant differences (at *p* < 0.05) between means.

### Declaration

We declare that plant materials were collected from private farms and permissions were taken from the owners following the relevant institutional, national, and international guidelines and legislation.

## Results

### Soil properties

The soil chemical investigation showed highly significant differences (*P* < *0.001*) in all chemical variables between contaminated and uncontaminated eggplant farms (Table 1). Soil pH, EC, and heavy metals were greater in the contaminated than in the uncontaminated soils, which contributed significantly higher macronutrients (N, P, and K) content. The heavy element's content of the contaminated soil was in the following order: Fe > Zn > Mn > Pb > Cu > Cd > Cr > Ni > Co > V. Furthermore, the PLI of the estimated heavy elements was greater than one, with Pb having the greatest value (248.9) and Ni having the lowest (1.0).

**Table 1:** Chemical characteristics (Mean ± SD) of contaminated and uncontaminated eggplant soils (*N* = *18*).

Soil variable		Uncontaminated sites	Contaminated sites	*t-*test	Permissible limit (WHO/FAO 2013)	PLI
pH		6.76 ± 0.20	7.54 ± 0.04	14.36***		
EC μS cm^−1^		2.15 ± 0.02	7.89 ± 0.03	32.26***		
Total N	mg kg^−1^	263.67 ± 37.67	67.19 ± 7.63	132.42***		
Total P		22.45 ± 1.5	6.64 ± 1.81	163.75***		
K		409.67 ± 58.36	32.09 ± 2.65	3.44.62***		
Pb		0.21 ± 0.01	51.86 ± 6.51	246.42***	0.01 – 50	248.9
Cd		0.80 ± 0.20	1.02 ± 0.04	62.37***	0.02 – 0.7	1.3
Cr		0.25 ± 0.10	0.81 ± 0.01	36.23***	5.00 – 30.00	3.3
Cu		1.49 ± 0.04	22.72 ± 2.68	162.36***	0.27 – 100	15.3
Ni		0.26 ± 0.02	0.59 ± 0.1	11.24***	5.00	1.0
Fe		10.40 ± 1.02	133.62 ± 15.96	42.98***	0.15 – 7.00	12.8
Mn		19.60 ± 2.03	52.31 ± 12.31	18.90***	20.00	2.7
Zn		2.94 ± 0.06	62.13 ± 11.53	72.38***	10.00 – 50.00	21.1
Co		0.19 ± 0.01	0.41 ± 0.02	9.32***	0.02	2.2
V		0.09 ± 0.01	0.24 ± 0.8	22.56***	0.001	2.6

***: p < 0.001.

### Growth measurements

**Figure 2:**
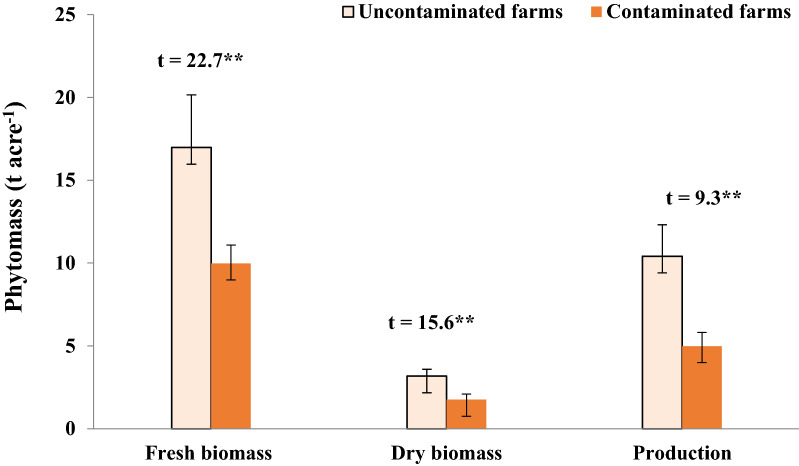
Biomass and production of eggplant crop grown in uncontaminated and contaminated farms. Vertical bars are standard deviation. **: p < 0.01.

In heavy metals-contaminated farms, eggplant growth measures revealed significant reductions in the root and shoot length, as well as the number of leaves (Table 2). The shoot length was significantly reduced from 94.1 ± 13.5 to 44.7 ± 9.7 cm, while the root length from 11.74 ± 1.75 to 6.4 ± 0.9 cm, and the number of leaves was from 44.7 ± 3.5 to 31.8 ± 2.7 leaves/individual under contamination conditions. In the same context, the dry and fresh biomass, and production of eggplants showed significant differences (*P* < 0.01) between uncontaminated and contaminated farms (Fig. 2). The mean fresh biomass, dry biomass, and production were significantly reduced from 16.9 ± 2.1, 3.2 ± 0.4, and 10.4 ± 1.9 to 9.9 ± 3.2, 1.8 ± 0.3, and 5.0 ± 0.8 t ha^−1^, respectively under contamination conditions with reduction percentages of 41.2, 44.6, and 52.1%, respectively.

**Table 2:** Growth characteristics (Mean ± SD) of the eggplants (*N* = 60) grown in contaminated and uncontaminated farms.

Variable	Farm		*t*-test	Reduction (%)
	Uncontaminated	Contaminated		
Density (individuals 100 m^−2^)	99.7 ± 18.1	98.3 ± 12.5	1.2	1.3
Stem length (cm)	94.1 ± 13.5	44.7 ± 9.7	5.1*	52.5
Root length (cm)	11.7 ± 1.8	6.4 ± 0.9	8.3**	45.2
Number of leaves/individual	44.7 ± 3.5	31.8 ± 2.7	4.4**	29.0

*p < 0.05, **: p < 0.01.

### Plant analysis

#### Plant pigments

**Figure 3:**
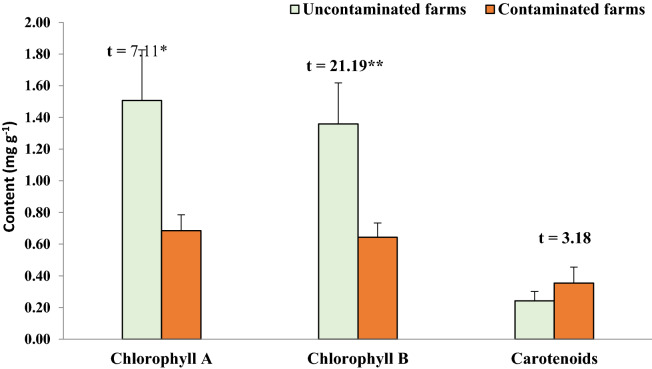
Photosynthetic pigments content of the eggplant leaves cultivated in uncontaminated and contaminated farms. Vertical bars are standard deviation. **: p < 0.01.

Chlorophyll a and b levels in eggplant leaves differed significantly between uncontaminated and contaminated plants (Fig. 3). Under the contaminated circumstances, chlorophyll a and b concentrations were declined from 1.51 ± 0.32 to 0.69 ± 0.10 mg g^−1^ and from 1.36 ± 0.26 to 0.64 ± 0.09 mg g^−1^, respectively. Meanwhile, the carotenoid content in the infected plants did not significantly increase from 0.24 ± 0.06 to 0.35 ± 0.10 mg g^−1^.

#### Plant nutrients

The nutrient analysis revealed considerable variations (*P* < *0.05*) among the various organs of eggplants from contaminated and uncontaminated fields (Table 3). The largest amounts of N, P, and proteins (1.98 ± 0.11, 2.08 ± 0.34, and 12.40 ± 0.67%) were observed in the shoots of uncontaminated plants, while the roots of the same plants had the highest K content (44.70 ± 0.88 mg kg^−1^) and the lowest P and carbohydrates (1.10 ± 0.13 and 13.90 ± 1.58%). Furthermore, the roots of the contaminated eggplants had the highest carbohydrate content (17.83 ± 1.90%) and the lowest N, K, and protein contents (1.15 ± 0.21%, 18.86 ± 1.14 mg kg^−1^, and 7.19 ± 1.31%).

**Table 3:** Nutrient constituents in the shoots and roots (*N* = *36*) of the eggplants grown in uncontaminated and contaminated farms. Maximum and minimum values are underlined.

Variable	Uncontaminated farm		Contaminated farm	
	Shoot	Root	Shoot	Root
**Inorganic**				
N (%)	1.98 ± 0.11a	1.20 ± 0.20b	1.22 ± 0.10b	1.15 ± 0.21b
P (%)	2.08 ± 0.34a	1.10 ± 0.13c	1.63 ± 0.20b	1.58 ± 0.16b
K (mg kg^−1^)	41.98 ± 0.72b	44.70 ± 0.88a	21.79 ± 0.68c	18.86 ± 1.14d
**Organic (%)**				
Carbohydrates (%)	14.61 ± 1.11b	13.90 ± 1.58b	17.57 ± 1.39a	17.83 ± 1.90a
Proteins (%)	12.40 ± 0.67a	7.52 ± 1.22b	7.61 ± 0.60b	7.19 ± 1.31b

Means with the same letters in the same raw are not significantly differed (Duncan’s multiple range tests at *P* < *0.05*).

#### Plant heavy metals

The examination of heavy metals in plant tissues revealed considerable variance (*P* < *0.05*) among the various organs of eggplants, with all analyzed metal contents being markedly higher in contaminated farms than in uncontaminated farms (Table 4). Furthermore, the belowground roots acquired larger metal concentrations than the aboveground shoots. The highest quantities of the studied heavy metals were found in the roots of contaminated plants, while the lowest contents of all metals were found in the shoots of uncontaminated eggplants, except for Zn (4.52 ± 1.03 mg kg^−1^). The contaminated plant roots accumulated heavy metals content (mg kg^−1^) in the order: Fe (2076.33 ± 43.02) > Mn (158.17 ± 13.84) > Zn (110.83 ± 15.69) > Ni (46.33 ± 10.61) > Cu (42.00 ± 12.56) > Cr (34.33 ± 9.29) > Cd (23.00 ± 4.27) > Pb (16.85 ± 1.22) > Co (4.53 ± 0.55) > V (0.42 ± 0.03), while the contaminated shoots was Fe (1836.83 ± 58.18) > Mn (67.67 ± 10.41) > Zn (49.00 ± 3.77) > Ni (26.21 ± 8.78) > Cr (19.17 ± 2.10) > Cd (15.33 ± 0.76) > Pb (14.00 ± 3.28) > Cu (13.67 ± 2.02) > Co (2.93 ± 0.13) > V (0.40 ± 0.05).

**Table 4:** Heavy metals concentration (Mean ± SD) in the shoots and roots (*N* = 36) of the eggplants cultivated in uncontaminated and contaminated farms. Maximum and minimum values are underlined.

Heavy metal (mg kg^−1^)	Uncontaminated farms		Contaminated farms	
	Shoot	Root	Shoot	Root
Pb	6.75 ± 2.18c	13.83 ± 3.01b	14.00 ± 3.28b	16.85 ± 1.22a
Cd	0.75 ± 0.25c	0.92 ± 0.58c	15.33 ± 0.76b	23.00 ± 4.27a
Cr	0.46 ± 0.05d	1.08 ± 0.38c	19.17 ± 2.10b	34.33 ± 9.29a
Cu	0.33 ± 0.14c	0.58 ± 0.14c	13.67 ± 2.02b	42.00 ± 12.56a
Ni	2.33 ± 1.81c	4.00 ± 0.75c	26.17 ± 8.78b	46.33 ± 10.61a
Fe	682.67 ± 26.55c	711.75 ± 16.27c	1836.83 ± 58.18b	2076.33 ± 43.02a
Mn	8.33 ± 1.18c	12.67 ± 0.63c	67.67 ± 10.41b	158.17 ± 13.84a
Zn	8.25 ± 0.9c	4.52 ± 1.03d	49.00 ± 3.77b	110.83 ± 15.69a
Co	0.82 ± 0.08d	1.47 ± 0.08c	2.93 ± 0.13b	4.53 ± 0.55a
V	0.03 ± 0.0b	0.04 ± 0.01b	0.40 ± 0.05a	0.42 ± 0.03a

Means with the same letter in the same raw is not significantly differed (Duncan’s multiple range tests at *P* < *0.05*).

Regarding the heavy metals’ analysis of the edible fruits of the eggplant, it was observed that all investigated metals were considerably (*p* < 0.01) higher in the contaminated than uncontaminated plants (Table 5). The order of heavy metals concentration accumulated by the contaminated fruits was Fe (2051.67 ± 2.89) > Mn (76.83 ± 0.29) > Zn (54.42 ± 0.08) > Ni (45.00 ± 0.5) > Cr (30.83 ± 0.29) > Pb (28.33 ± 0.76) > Cd (23.83 ± 0.29) > Cu (19.67 ± 0.29) > Co (2.93 ± 0.03) > V (0.18 ± 0.01), while that of the uncontaminated fruits was Fe (95.47 ± 7.08) > Mn (25.12 ± 3.59) > Cr (10.29 ± 1.28) > Zn (5.36 ± 0.13) > Pb (4.91 ± 0.14) > Ni (3.13 ± 0.11) > Co (1.17 ± 0.29) > Cu (0.48 ± 0.01) > Cd (0.31 ± 0.01) > V (0.03 ± 0.01).

**Table 5:** Heavy metals concentration (Mean ± SD) in the edible fruits (*N* = *18*) of the eggplants cultivated in uncontaminated and contaminated farms.

Heavy metal	Farms		t-test
	Uncontaminated	Contaminated	
Pb	4.91 ± 0.14	28.33 ± 0.76	46.91***
Cd	0.31 ± 0.01	23.83 ± 0.29	134.43***
Cr	10.29 ± 1.28	30.83 ± 0.29	9.03**
Cu	0.48 ± 0.01	19.67 ± 0.29	112.19***
Ni	3.13 ± 0.11	45.00 ± 0.50	122.71***
Fe	95.47 ± 7.08	2051.67 ± 2.89	48.65***
Mn	25.12 ± 3.59	76.83 ± 0.29	10.72**
Zn	5.36 ± 0.13	54.42 ± 0.08	1057.34***
Co	1.17 ± 0.29	2.93 ± 0.03	10.86**
V	0.03 ± 0.01	0.18 ± 0.01	179.00***

**: p < 0.01, ***: p < 0.001.

#### Transfer factors (TF)

The eggplants' ability to accumulate heavy elements in polluted soils was revealed by the descriptive statistical test represented by the transfer factor (Table 6). The TF of all examined heavy metals (excluding Pb and Zn) from soil to plant root exceeded 1, with Ni having the greatest value (201.43). It was arranged as Ni > Cr > Cd > Fe > Co > Cu > V > Mn > Pb > Zn. On the other side, the TF of all determined metals, except Mn and Zn (1.16 and 4.52), from the plant root to the shoot was less than 1. Moreover, the TF of Pb, Cr, Mn, and Zn (1.02, 5.21, 1.65, and 3.11) from the plant root to the fruits of the eggplant was greater than 1, while that of the other investigated metals did not exceed 1.

**Table 6:** Transfer factor (TF) of heavy metals from the soil to the different organs of the eggplants grown on uncontaminated and contaminated farms. Values > 1 are bold.

Heavy metal	Transfer factor		
	Root	Shoot	Fruit
Pb	0.32	0.66	**1.02**
Cd	**22.55**	0.74	0.69
Cr	**42.38**	0.49	**5.21**
Cu	**1.85**	0.45	0.65
Ni	**78.53**	0.57	0.88
Fe	**15.54**	0.92	0.56
Mn	**1.11**	0.91	**1.65**
Zn	0.17	**3.17**	**3.11**
Co	**11.05**	0.60	0.72
V	**1.75**	0.85	0.55

#### Health risk assessment

The safety of eggplant consumption was assessed using the health risk index (HRI) as the heavy metals safety index, which requires at first the determination of the DIM for children and adults consumers (Table 7). The DIM of the estimated heavy elements (except Mn in contaminated farms) by consuming eggplants was less than one for both adults and children. For instance, the DIM of Mn was 1.08 and 1.24 mg day^−1^ for adults and children, respectively. Moreover, the results of the health risk assessment using the HRI showed the presence of health risks from consuming eggplants in the uncontaminated sites due to Pb (HRI: 2.57) for adults and Pb and Mn (2.96 and 1.08) for children. Besides, there are health risks from consuming the contaminated eggplants due to the high HRI of Pb, Cd, Ni, Fe, and Mn for adults (14.86, 12.50, 1.18, 1.54, and 2.88, respectively), and children (17.09, 14.37, 1.36, 1.77, and 3.31, respectively).

**Table 7:** Individual heavy metals in the edible sections of eggplants planted in uncontaminated and contaminated soils using the daily intake of metals (DIM: mg day1) and the health risk index (HRI) of adults (A) and children (C).

Heavy metal	Uncontaminated sites				Contaminated sites				R_f_D	References
	DIM		HRI		DIM		HRI			
	A	C	A	C	A	C	A	C		
Pb	0.003	0.003	**2.57**	**2.96**	0.02	0.02	**14.86**	**17.09**	1.0 × 10^–3^	(US-EPA 2013)
Cd	0.000	0.000	0.16	0.19	0.01	0.01	**12.50**	**14.37**	1.0 × 10^–3^	(US-EPA 2013)
Cr	0.000	0.000	0.07	0.08	0.00	0.00	0.21	0.25	1.5 × 10^0^	(US-EPA 2013)
Cu	0.005	0.006	0.00	0.00	0.02	0.02	0.01	0.01	4.0 × 10^–2^	(FAO/WHO 2013)
Ni	0.000	0.000	0.04	0.01	0.01	0.01	0.26	0.30	2.0 × 10^–2^	(US-EPA 2010)
Fe	0.002	0.002	0.08	0.09	0.02	0.03	**1.18**	**1.36**	7.0 × 10^–1^	(FAO/WHO 2013)
Mn	0.050	0.058	0.07	0.08	**1.08**	**1.24**	**1.54**	**1.77**	1.4 × 10^–2^	(FAO/WHO 2013)
Zn	0.013	0.015	0.94	**1.08**	0.04	0.05	**2.88**	**3.31**	3.0 × 10^–1^	(FAO/WHO 2013)
Co	0.003	0.003	0.01	0.01	0.03	0.03	0.10	0.11	4.0 × 10^–2^	(US-EPA 2013)
V	0.001	0.001	0.01	0.02	0.002	0.002	0.04	0.04	1.8 × 10^0^	(FAO/WHO 2013)

## Discussion

Heavy metals contamination of the agricultural soils is a severe worry for food safety and a potential health hazard, in addition to its impact on the soil ecosystem^37,38^. The contaminated soils had higher pH, EC, and heavy metal values than the uncontaminated soils, resulting in significantly higher macronutrient content. These results agreed with those of Galal^39^, Eid et al.^40^, and Shehata and Galal^25^. The elevated heavy metal contents in the contaminated sites are the result of the industrial effluents, municipal wastewater, and the excessive use of pesticides and fertilizers. Galal et al.^41^ reported similar findings in south Greater Cairo. According to WHO/FAO^42^, soil Pb, Cd, Fe, Mn, Zn, Co, and V of the contaminated sites and soil Cd, Fe, Co, and V of the uncontaminated site exceeded the permissible limits for normal soils. Moreover, the PLI data revealed that the contaminated soils were highly polluted (PLI > 5) with Pb, Cu, Fe, and Zn; moderately polluted (PLI = 1–4) with Cd, Cr, Mn, Mn, Co, and V; and unpolluted (PLI < 1) with Ni^31^. Similar output was postulated by Galal et al.^16,41,43^.

The growth properties of the eggplant recognized significant reductions in the measured parameters except for plant density in heavy metals-contaminated farms. Under contamination, the length of the shoot and root, besides the number of leaves, declined by 52.5, 45.2, and 29.0 percent, respectively. These findings corroborated those of Chaturvedi et al.^44^ and Ai et al.^45^, who found that eggplant growth prevention is a prevalent indication of heavy metal stress. Additionally, Ekmekçi et al.^46^ reported a 19.2% reduction in the eggplant height under heavy metals stress. The growth reduction of the eggplant may be due to its uptake of high contents of heavy elements such as Ni, Cu, Fe, Mn, Cr, Cd, and Pb in its different tissues. Plant growth reduction due to heavy metals was reported by several researchers^16,25,40,43,47^, especially Pb and Cd, which significantly reduce root and stem length beside the number of leaves of the eggplant^48^. Besides, Farahat et al.^49^ and Ghazi et al.^50^ reported that Co and Cr ions inhibit shoot and root length, shoot and root biomass, and the number of leaves, and attributed this inhibition to the reduction in cell elongation and cell division, photosynthetic pigments, and photosynthetic activity. Moreover, the eggplant responds to heavy metals, especially Cu stress through the prevention of the shoot and/or root growth^44,51^. Plants may withstand heavy metal stress by accumulating these metals in their roots and preventing their transfer to other sections of the plant^52^. Consequently, heavy metals stored in the root inhibits its growth rather than affecting other organs.

Generally, the present study observed a considerable decrease in the eggplant biomass and production in heavy metals-contaminated farms. Under contamination conditions with significant heavy metals concentrations, the eggplant's fresh and dry biomass and production were reduced by 41.2, 44.6, and 52.1%, respectively. The results of Ai et al.^53^ recorded a 35.84% reduction in eggplant production in wastewater-irrigated soils. Vecchia et al.^54^ attributed the decrease in plant growth to the prevention of mitotic division caused by Pb and Cd heavy metals. Additionally, Rizwan et al.^55^ reported a negative impact of Cd on the biomass and yield of various vegetables. Also, essential elements (such as Zn and Cu) are necessary for plant development, but excessive amounts have a negative impact^56^. According to Ai et al.^53^, a combination of Cu, Zn, Pb, and Cd significantly affects eggplant biomass and production. On the other side, the high biomass and production of the eggplant in the uncontaminated farms may be attributed to the high nutrients (N, P, and K) content. This result coincided with Eid et al.^40^ who attributed the high yield of kidney beans to the higher contents of soil macronutrients and organic matter. Moreover, increasing N content can promote the growth of aboveground shoots resulting in high biomass and production^45^.

Chlorophyll a and b are required for photosynthesis and are very susceptible to heavy metal pollution stress^46^. Plants with high metal toxicity have a variety of growth disorders, including photosynthesis^51^. The chlorophyll a and b levels in plants cultivated in contaminated soil decreased significantly, which could be due to eggplant's high absorption capability for heavy metals that impede chlorophyll production^32,41^. Additionally, the high salinity of the contaminated sites enhances chlorophyllase activity, which degrades plant chlorophylls^16^. Chaturvedi et al.^44^ found a negative relationship between heavy metals quantity and pigments content in eggplant leaves. Furthermore, Piotrowska et al.^57^ and Singh et al.^58^ hypothesized that heavy metals like Pb could impede the biosynthesis of chlorophyll by blocking the intake of necessary components for photosynthetic pigments (e.g., Mg, K, Ca, and Fe). This may interpret the lower contents of macronutrients in the tissues of eggplant cultivated in the contaminated sites. According to Collado-González et al.^59^, macronutrients such as N, P, and K enhance the production of cauliflower curds. However, Kumar et al.^11^ stated that the high content of macro-and micro-nutrients may induce crop yield but may also negatively impacts biological activity. Moreover, the eggplant highly uptake K because it is a principal nutrient for its development^17^.

Compared to normal soils, vegetable plants absorb and store more heavy metals from contaminated soils^60^. In a similar trend, the current study found that eggplants grown in contaminated farms collected much greater levels of heavy metals than eggplants grown in uncontaminated fields. In the same context, Galal et al.^16^ and ur Rehman et al.^61^ stated that crop plants uptake higher heavy metal concentrations from wastewater-irrigated than freshwater-irrigated soils. Besides, the belowground roots accumulated higher concentrations of all investigated metals than the aboveground shoots. Tolerant plants, such as eggplant, tend to reduce soil-root and root-shoot transfer, resulting in less biomass accumulation^41^. The heavy metal contents in eggplant fruits were lower than in green vegetables like cabbage and common mallow^16,43^. According to Chowdhury et al.^7^, heavy element deposition in leafy vegetables is greater and easier than in fruit or grain crops, even though ingestion of these plants is the principal avenue of human exposure to heavy metals. It’s worth noting that the quantities of Cu, Ni, Pb, Cd, and Co in the shoots and edible fruits of eggplant cultivated in contaminated soils, as well as shoot Cr and fruit Mn, were above the permissible range for normal plants^29,52,62^. As reported by Ai et al.^45^, solanaceous edible fruits were mostly polluted with Pb, while the Cd concentration surpassed the standard level, exclusively in eggplant. They also found that Cu, Zn, and Pb levels in eggplant fruits were higher than the National Food Safety Standard for Contaminants in Foods (GB2762-2017). The heavy metal contents in the edible sections of eggplant grown in contaminated fields were higher than those found by Jolly et al.^63^, Zhou et al.^64^, and Chaturvedi et al.^44^ on the same plant grown in contaminated soils.

Heavy metals translocation is a metabolic pathway that is primarily affected by chemical and physical factors^44^. They could be taken up by eggplants from the soil, accumulated in their roots, and possibly transferred to their edible fruits. The transfer potential of eggplants revealed that, except for Pb and Zn, they primarily retain the heavy metals studied in their subterranean organs rather than translocating them to the aerial parts. This result can be related to the poor mobility of these metals from below-ground to above-ground components, making this plant a good choice for the phytostabilization of these metals. In the contaminated farms, the TF of the studied heavy elements from soil to eggplant roots fell in the order: Ni > Cr > Cd > Fe > Co > Cu > V > Mn > Pb > Zn, which is like that reported by Ai et al.^53^ on eggplant, Shehata and Galal25 on cucumbers, Galal et al.^41^ on cauliflower, and Galal et al.^16,43^ on cabbage and common mallow, respectively. On the other hand, the eggplants had the potential to translocate Mn and Zn to their shoot and Pb, Cr, Mn, and Zn to the edible fruits. These results indicate the possibility of eggplant being an accumulator and phytoextractor of these metals. Chaturvedi et al.^44^ recorded that Pb can be translocated to the shoots, but not to the fruits, while Cd was stored in the roots of eggplant. The translocation and accumulation of these metals in the cauliflower’s edible section will expose the public consumers to high health risks.

The nutritional quality of vegetables is a great concern since vegetables are a main component of the human diet^8^. Heavy metals accumulation in the edible organs of vegetable plants is a common hazard to the consumers’ health^61^. The DIM for the studied heavy metals (except Mn in contaminated farms) by consuming eggplants was less than one for both children and adults. For instance, the DIM of Mn was 1.24 and 1.08 mg day^−1^ for children and adults, respectively. These results coincided with those of Chaturvedi et al.^44^ on contaminated eggplant, Rehman et al.^65^, and ur Rehman et al.^61^ on wastewater-irrigated cauliflower. Moreover, the HRI showed the presence of health hazards from consuming eggplants in the uncontaminated sites due to Pb for adults and Pb and Mn for children. Besides, there are health risks from consuming the contaminated eggplants due to the high HRI (> 1) of Ni, Fe, Mn, Cd, and Pb for children and adults. In a corresponding study, Saeedifar et al.^66^ and Ai et al.^45^ found that the accumulation of Pb and Cd negatively affects the safety of eggplant fruits. According to the US-EPA35, the HRI > 1 of these metals shows that consumers are exposed to health risks because of the eggplant's large proportion in local populations' diets, posing a greater danger to human health. Galal et al.^41^ also observed health problems from eating contaminated cauliflower due to excessive dietary Pb, Cd, Mn, Fe, and Ni levels. Khanal et al.^67^ and Ma et al.^13^ recorded similar findings when it came to the health effects of these metals when eating contaminated cauliflower.

## Conclusion

The contaminated soils supporting eggplant growth were highly polluted with Fe, Cu, Pb, and Zn, and relatively polluted with Cd, Cr, Mn, Mn, Co, and V. Cultivation of eggplant in contaminated soils can result in high amounts of Cu, Co, Ni, Mn, Cd, and Pb in its edible sections, exceeding the permissible level for normal plants, and exposing public consumers to significant health risks. The heavy metal contamination stress lowered morphological measures and photosynthetic pigments, which in turn affected eggplant crop growth and production. The eggplant retained most investigated heavy metals (except Pb and Zn) in its root due to their low mobility, and consequently, this plant is an appropriate candidate for these metals’ phytostabilization. However, the study plant had the potential to translocate Mn and Zn to its shoot and Pb, Cr, Mn, and Zn to the edible fruits indicating its possibility to be a phytoextractor and accumulator of these metals. The present study indicated health risks from consuming the contaminated eggplants due to the dietary intake of Fe, Mn, Cd, Ni, and Pb for adults and children. Therefore, environmental protection laws must be applied to control industrial and municipal wastes discharged into the agricultural lands and hence avoid the entrance of toxic metals into the food chain. Besides, washing eggplant with water and various household chemical solutions can significantly remove heavy metals. For safety issues and food sustainability, our investigation strongly recommends the cultivation of eggplant in uncontaminated areas and avoid, as possible, its cultivation in contaminated agricultural lands due to its toxic effects even in the long run.

## Data Availability

All data generated or analyzed during this study are included in this published article.
